# The feasibility of a crowd-based early developmental milestone tracking application

**DOI:** 10.1371/journal.pone.0268548

**Published:** 2022-05-26

**Authors:** Ayelet Ben-Sasson, Kayla Jacobs, Eli Ben-Sasson

**Affiliations:** 1 Occupational Therapy Department, Faculty of Social Welfare and Health Sciences, University of Haifa, Haifa, Israel; 2 Faculty of Computer Science, Technion, Haifa, Israel; 3 StarkWare Industries Ltd, Netanya, Israel; All India Institute of Medical Sciences - Bhopal, INDIA

## Abstract

**Objective:**

Parents’ tracking of developmental milestones can assist healthcare providers with early detection of developmental delays and appropriate referrals to early intervention. Crowdsourcing is one way to update the content and age data distribution of developmental checklists for parents and providers. This feasibility study examined which developmental milestones parents chose to track and what they added beyond traditional milestones, using the babyTRACKS crowd-based mobile app.

**Method:**

We analyzed the developmental diaries of 3,832 children, registered in the babyTRACKS app at an average age of 9.3 months. Their parents recorded a median of 5 milestones per diary, selecting from the accumulating lists of age-appropriate milestones or authoring new milestones. The final database included 645 types of milestones; 89.15% were developmental, of which 43.6% were comparable to the Centers for Disease Control (CDC) milestones while the rest were crowd-authored. Milestones were categorized into developmental domains: Gross Motor, Fine Motor, Oral Motor, Self-Care, Cognitive, Language Comprehension, Speech, Non-Verbal Communication, Social, Emotional, and Regulation.

**Results:**

On average, the milestone domains of Gross Motor, Fine Motor, Cognitive and Social were the most added to diaries (20%-30% of a diary). Within the Cognitive, Speech and Language Comprehension domains there were significantly more CDC comparable versus crowd-authored milestones (29% versus 21%, 22% versus 10%, 8% versus 4%). In contrast, within the Regulation and Oral Motor domains there were more crowd versus CDC milestones (17% versus 3%, 9% versus 3%). Crowd-authored Speech milestones were significantly older by 7 months than CDC milestones.

**Conclusion:**

Tracking daily observations of child development provides a window into personally relevant milestones for the child and parent. The crowd of parents can independently track and add new milestones across main developmental domains. Regulation and Oral Motor development especially interest parents. Parents may be less aware of early progress in Language Comprehension and Speech; thus, these domains require more structured screening. Designing mobile early screening which is crowd-based engages parents as proactive partners in developmental tracking.

## Introduction

Early developmental surveillance assists in detecting delays in children and facilitates parents’ awareness of expected developmental milestones [1]. The earlier a delay is detected, the greater the chances of maximizing outcomes of early intervention [2]. Unfortunately, a significant proportion of children with developmental delays do not receive early intervention [3]. Barriers to routine healthcare screening include limited health system resources, insufficient training, and knowledge among primary care providers, as well as inaccessibility to healthcare services in underserved communities [3–7]. In addition, the evidence base [1] and ecological validity [8] of the tools providers use vary extensively. Parents have more opportunities than providers do to observe their children’s behavior [9] and are often the first to notice problems [10]. Hence, recruiting parents as partners in monitoring child development can advance screening efforts. Mobile applications offer an opportunity for recruiting parents as they are already sharing personal observations regarding their child on social media [11, 12]; daily observations that can be valuable for early detection.

babyTRACKS (formerly known as Baby CROINC) is a free, crowd-based, mobile application to track early childhood development. It enables parents to record their child’s milestones as they emerge and compare with other children [13]. The platform aims to increase parents’ awareness of typical development, lead to earlier detection of developmental delays, and improve partnership with healthcare providers [13–15]. This study examines which developmental milestones babyTRACKS parents chose to track and what they added beyond the milestones found in existing, traditional developmental checklists. Designing an open, parent-driven application in which parents select when and what aspects of their children to track, was founded on the premise that each child develops uniquely as a product of their personal abilities, environmental opportunities and demands [16, 17]. Thus, the milestone database described in this paper reflects the notion that crowd-generated milestones mirror individualized facets of development as well as the behaviors of greatest interest, concern, and noticeability to parents.

### Traditional screening

Early developmental screening is primarily driven by experts who determine whether children meet a specific set of milestones at particular ages. While clinical guidelines recommend the use of standardized, evidence-based screening tools during visits [18, 19] in practice many providers rely on an informal milestone history [1, 3, 5, 20]. Providers can use questionnaires and published developmental checklists. Examples of popular developmental screening tools are the ASQ-3 [21] and PEDS-DM [22] norm-referenced parent questionnaires. These questionnaires assess a closed set of behaviors at each age range. While a “one-size” model simplifies administration and interpretation of data, it is not adaptable for individual contexts.

In contrast to the above questionnaires, developmental checklists present providers with an expected list of milestones for each age group and vary in their evidence base [1]. One prominent checklist by the United States Centers for Disease Control and Prevention (CDC) [23] was compiled based on two child development textbooks [24, 25]. The CDC outlines expected milestones within the Motor, Cognitive, Social-Emotional and Language-Communication domains for 10 age groups, from 2 to 60 months. Wilkinson et al. [26] evaluated the concordance in content and age ranges listed in the CDC checklist relative to three other checklists. Findings demonstrated that only 17.9% of milestones overlapped in at least three of the checklists and that 26.9% of these overlapping milestones were associated with different age ranges. Most of the identified overlapping milestones related to the motor domain. This suggests lower consensus with core milestones in non-motor domains. Furthermore, the milestone ages clinicians rely upon for Communication, Cognitive, and Social-Emotional milestones are often based on a small sample size or on charts in textbooks that do not provide percentiles [1]. As such, reliance on these for developmental check-ups does not consider the typical distribution around a milestone age [27].

### Digital screening

The need to integrate low-cost, accessible, data-based means for routine healthcare screening can be met by engaging parents to record their children’s developmental progress online. Most digital screening tools are web-based versions of existing screening tools that parents complete upon provider referral. Research supports the comparable validity of these web versions to traditional paper-and-pencil versions (e.g., [28–30]). In a recent review of web-based screening tools research [31] the majority used web versions of screening tools for autism spectrum disorder, language, and motor delays. Designing digital platforms for general child development tracking can serve a broader range of needs and indicate specific areas that require more in-depth assessment.

There are digital platforms which adapt screening tools into user-friendly, online versions. The Baby Steps platform sends text messages to parents with age-relevant ASQ-3 milestones to report upon. Evidence supports its feasibility and acceptability for low-income mothers [32, 33]. Similarly, research shows the feasibility of another application that sends milestone questions from ASQ to low-income mothers, with close support of providers [34]. Both platforms are examples of highly structured tools monitored by experts, which are costly when thinking of designing universal digital screening tools.

### Crowdsourcing and developmental tracking

Crowdsourcing child development tracking offers a way to update and scale the database of developmental milestones to be meaningful for parents and providers to monitor. Crowdsourcing involves gathering input from a large pool of people to collectively solve a problem or task [35]. This method helps lower task costs and increases efficiency, and leads to a large diversity and scale of inputs. Crowdsourcing has been used in health-related areas of genetics, psychiatry, epidemiology, and nutrition [35], though only rarely in pediatrics (1% in [36]). One of the challenges of crowdsourcing health tasks is balancing between the quantity and quality of the incoming data. The data complexity and problem solving involved in many clinical crowdsourcing applications requires input from clinical experts to assist in processing crowd data that can increase the utility and validity of the data [36]. The public can be involved in providing health data and/or in generating ideas [36, 37]. In a study of idea management in 166 online communities, 27% of users were active and 10% had more than one idea. Although not all users create new ideas, all users benefit from novel inputs. The current study relies on parents as the crowd for providing data, with some parents also generating new ideas for milestones.

Although early screening is recognized as an essential component of pediatric healthcare, in practice there are many barriers to early screening. Digital platforms can facilitate early detection by harnessing parent wisdom to track contemporary and context-relevant indices. Gathering parents’ unique observations about their child’s development can help providers better understand a child’s needs and tailor the visit to follow up on these observations. This study addressed the following research questions: (1) What types of developmental milestones do parents record for their child? (2) How do developmental milestone concepts, domains, and ages created by the crowd of parents differ from CDC milestones? (3) What do parents record beyond developmental observations?

## Methods

**Fig 1:**
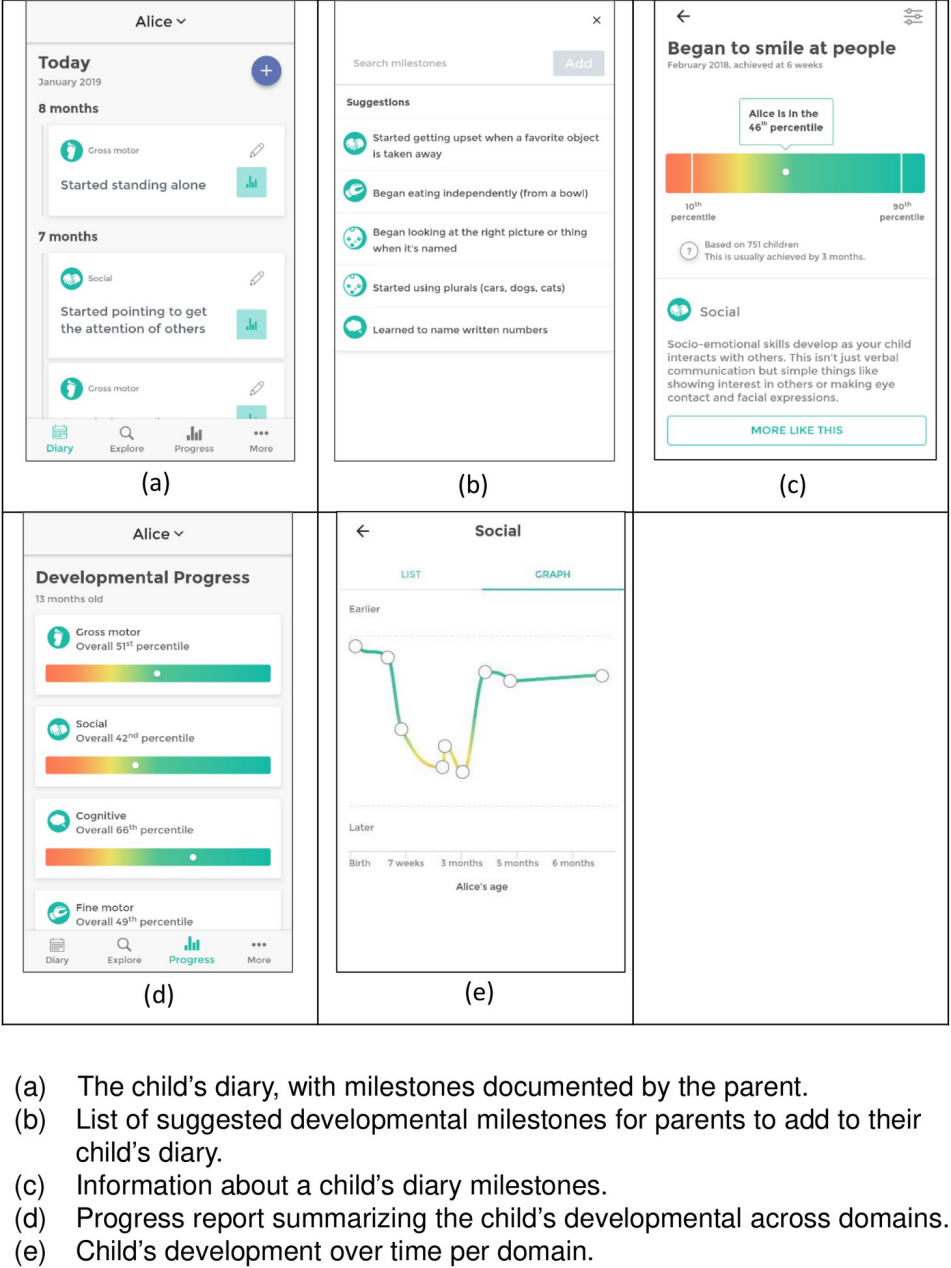
Screenshots of the babyTRACKS app.

babyTRACKS is a free mobile application parents can use to track their child’s early development. Parents download the app from the Android or Google store and create an account, signing a research consent form during this process as approved by the university’s Ethics Committee. Once parents open an account they are notified by “All set! Welcome to [*Name of child]*’s diary—here you can keep track of progress and development.” To build their children’s diaries, parents add age-dated milestones such as “began to smile at people” at 4 weeks, and “she blows kisses” at 12 months. Fig 1 presents screenshots from a sample diary.

Parents can create milestones in several ways: (1) entering an original milestone text, (2) searching for a specific keyword and selecting an existing milestone others added to their diary by using “autocomplete,” and (3) selecting milestones from lists suggested to the user on various app pages, such as the Explore Database page and the Add Milestone page, with the top 5 age-appropriate suggestions in the database at the time of the user’s app activity. The two latter methods (2, 3) accumulate statistics automatically, while the first method requires clinical expert management, as outlined below.

The system presents parents with statistical information related to the child’s milestone achievements in the form of percentiles (i.e., higher percentile implies earlier development relative to other children), for both individual milestones and for developmental domains. A percentile is computed relative to other children (only if the milestone was added to at least 10 children’s diaries) from the ranking of all ages reported for that milestone within babyTRACKS. Across a developmental domain, median percentiles are computed instead of means, so that this summary percentile would be insensitive to extreme outliers amongst the individual milestone percentiles. Domain percentiles reflect the ranking of all the child’s milestone percentiles within a domain.

babyTRACKS involves an expert curation process known as Crowd-Curated Intelligence (CCI) described in greater detail elsewhere [15]. Behind the scenes, child development experts manage new milestone texts by merging semantically similar, though linguistically distinct, milestone texts into unified milestone concepts (e.g., ‘Dan sat alone’ is merged with the existing concept ‘Began to sit without support’). Experts are notified by the system that a new text milestone was entered, and its details are managed on a dedicated database platform. In this way, information from multiple children about the same concept are aggregated to provide statistical comparisons. However, if the expert identified an incoming milestone that had no similar concept in the database, the curator creates a new, unique concept for that milestone (e.g., ‘Swiped phone for the first time’), which henceforth is suggested to parents, appears in database searches, and starts to accumulate statistics.

The CCI method also involves categorizing milestone concepts as associated with one or more developmental domains. This enables parents to view their child’s progress within five domains: Gross Motor, Fine Motor, Language Comprehension, Cognitive and Social. In addition, a more refined categorization into 11 domains was conducted for research purposes: Gross Motor, Fine Motor, Oral Motor, Speech, Language Comprehension, Cognitive, Non-Verbal Communication, Social, Emotional, Regulation, and Self Care.

### babyTRACKS reliability and validity evidence

The system was launched with 252 milestone concepts based upon milestones for the first five years of life published by the CDC [23]. Past babyTRACKS research showed that 8% of the children’s diary milestones were added as original parent-authored texts. However, 83% of these milestones were syntactic variations of existing milestones concepts (e.g., “Jenny began to toddle” compared to the existing “started walking” concept), while only 7% were semantically novel milestones that did not exist in the original database [15]. Research indicates that there has been a steady growth in the number of semantic novelties introduced by parents since the original milestone set, while continuing to gather/provide meaningful new knowledge [13]. The current study focuses on questions related to the developmental content of the milestones added to the database by parents relative to published core milestones.

The content validity of the database was established in a previous study [15]. This involved three child development experts, over 15 years of clinical experience working in early child development settings (e.g., developmental psychologist, occupational therapist), who independently judged the degree of importance (from “very unimportant” to “very important”) of a subset of 300 babyTRACKS milestones for assessing child development. They rated 62%–93% of the milestones as “important” or “very important” in evaluating a child’s developmental progress, as opposed to superfluous information [15]. The research described in this manuscript aimed to understand the novelties of crowdsourced data compared to published milestones, as well as the areas and ages that parents chose to track.

The categorization task was manualized, so that clinical experts worked with a guide that contained definitions and examples for each domain. After initial reliability was obtained, two experts in child development continued to review all categorizations and conflicts regarding curation. The CCI method was found reliable through comparing the classification of incoming milestones by two of the team members with extensive clinical pediatric experience and reaching at least 90% agreement.

In addition, a study was conducted to test the potential for semi-automatizing the CCI method. To this end, parents of young children without a professional background in a relevant field, simulated the CCI process while determining whether 100 milestones were new or similar to existing milestones. Parents reached high agreement (76%) with babyTRACKS’ categorization of these milestone texts [15].

The current study primarily aimed to understand the types of novel milestones that parents added to the database relative to the traditional CDC milestones.

### Database

**Fig 2:**
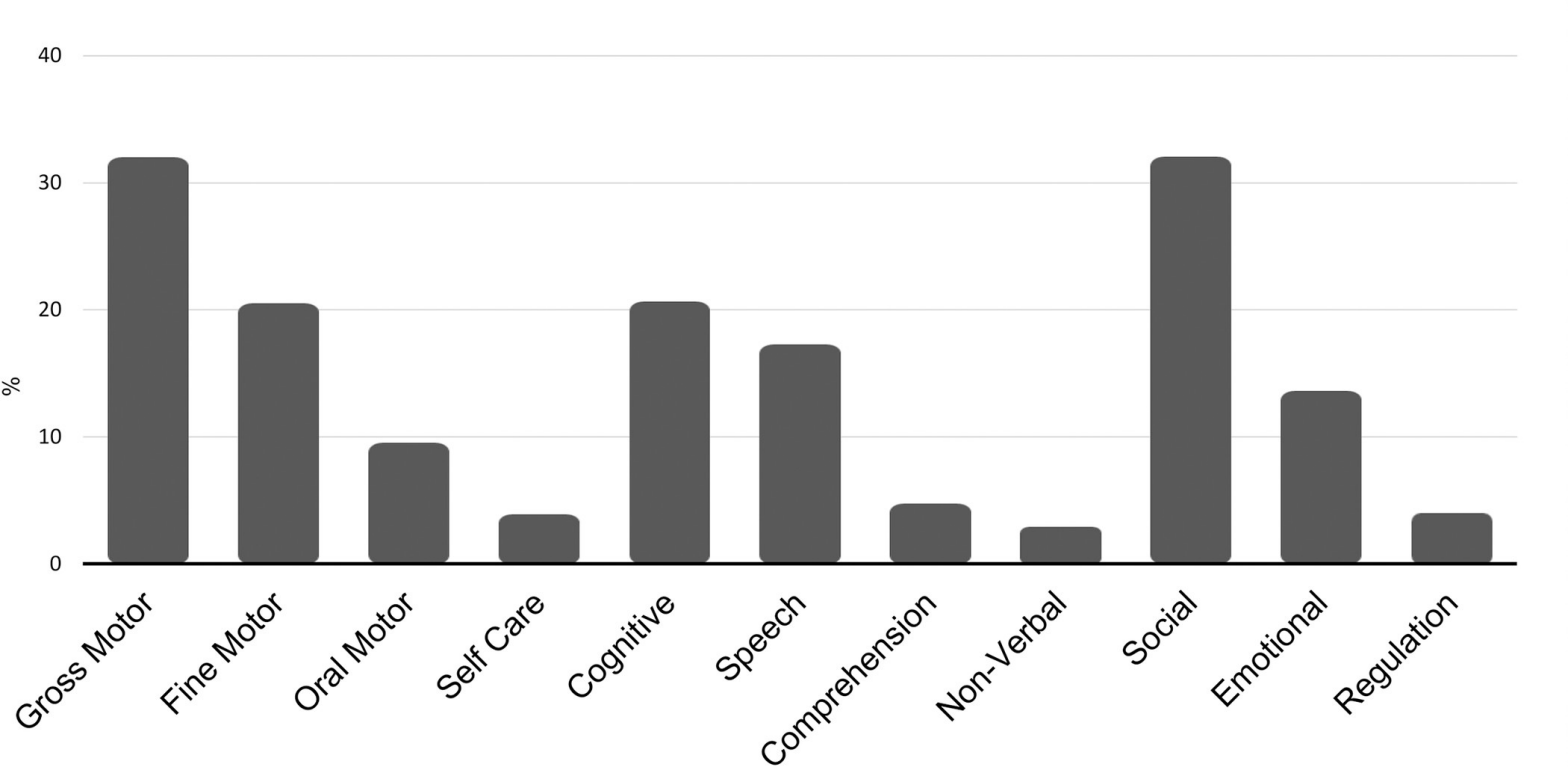
Average percent of milestones in each domain, in a diary. Note: These percentages do not add up to 100% as a diary milestone can be associated with more than one domain.

The babyTRACKS database reflects 3,832 diaries started between December 2014 and April 2020. Children’s ages at registration were an average of 9.33 months (Mdn = 4.75, SD = 12.96). Average weeks of pregnancy was 38.50 (SD = 2.38), and 51.6% of children were males. Diaries had a median of 5 milestones (M = 9.56, SD = 16.46, range 1–348). Most of the users were mothers (95%). For users with their country registered (n = 1,842), the eight top countries were Israel (26.38%), the USA (16.23%), India (8.6%), United Kingdom (7.44%), South Africa (5.16%), Philippines (4.92%), Australia (4.67%), and Canada (4.13%). The remaining 413 diaries were dispersed across 91 countries. Users added milestones to the diary in an average of 1.78 sessions (Mdn = 1, SD = 2.99). A session was defined as a period of user activity with up to a gap of an hour between actions. Fig 2 presents the average percent of milestones within each domain in a diary.

**Fig 3:**
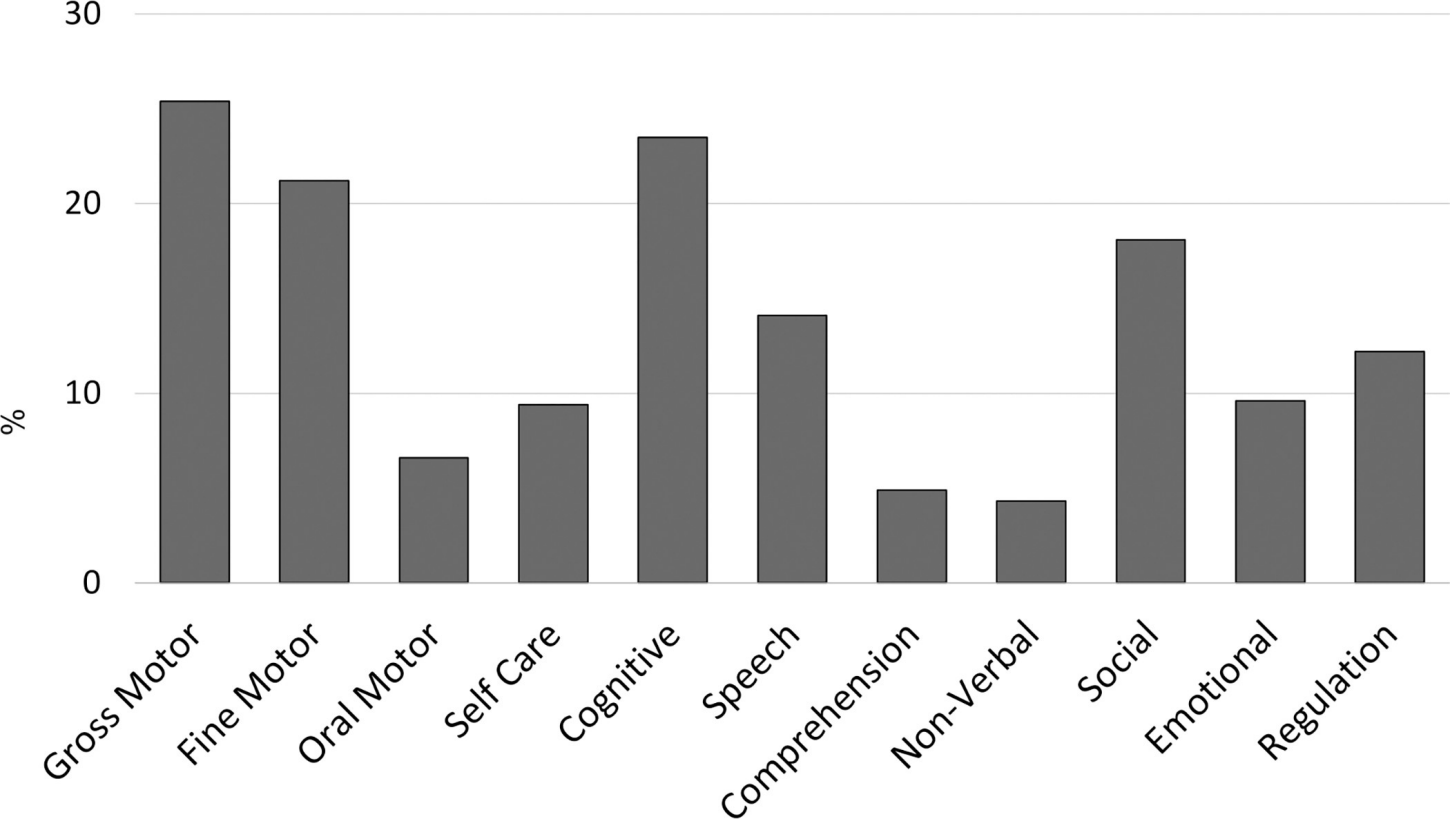
Percentage of milestones within the developmental domain in babyTRACKS database.

**Fig 4:**
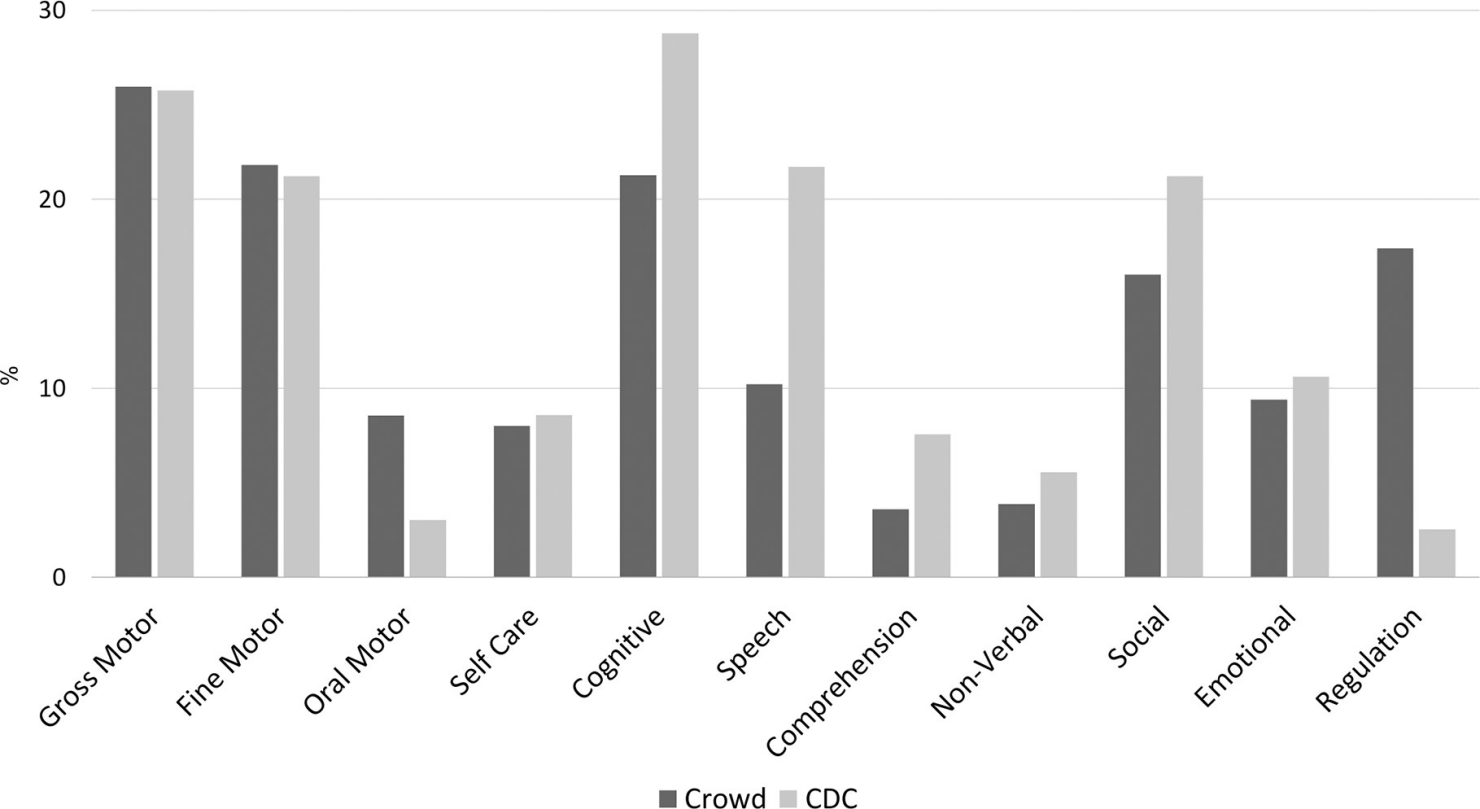
Percentage of milestones per domain within CDC versus crowd-based milestones.

This study focused on 675 milestone concepts, which included 34,390 individual diary milestones. A milestone concept had a median of 3 unique milestone texts associated with it. Milestone concepts analyzed only those that had at least one child associated with them (n = 46 were excluded for this reason due to the user deleting them from diary or deleting the diary). All nonsense/gibberish milestones were excluded as well. There were 575 (89.15%) developmental milestone concepts, of which 199 (34.61%) were comparable to CDC milestones. The endorsement of developmental milestones varied greatly (M = 59.66, Mdn = 15, SD = 106.46). See Fig 3 for the distribution of developmental domains within the database; see Fig 4 for the distribution of milestone concepts between developmental domains for CDC comparable and crowd-authored milestones. Note that 41.04% of the developmental milestones were associated with more than one domain. Domains with over 30% overlap were: 52% of Self Care milestones were associated with the Fine Motor domain; 48% of the Social milestones were associated with the Non-Verbal domain; 37% of Speech milestones were associated with the Cognitive domain; and 31.58% of Oral Motor milestones were associated with the Fine Motor domain.

### Data analysis

SPSS 25 was used for statistical analysis. Fisher’s exact tests were applied to compare the rate of milestones classified as CDC comparable versus crowd-authored within each domain to examine whether parents tend to author new milestones in certain domains. To investigate whether parents author milestones which are more complex in nature (i.e., record real-world behaviors) than traditional milestones, we conducted a Fisher’s exact test to compare the rate of milestones associated with multiple developmental domains versus one domain. Furthermore, we wanted to examine whether crowd-authored milestones differ in their age range and median from CDC comparable milestones overall and within each developmental domain using Mann Whitney U tests.

## Results

### Descriptives by type of milestones

Table 1 presents characteristics of the 10 most endorsed crowd-authored milestones and the most endorsed CDC-comparable milestones. See S1 Table for a list of app milestone examples by age group.

**Table 1:** Characteristics of the top 10 CDC milestones and top 10 crowd milestones in the app.

		Age (Months)				Developmental Domain(s)
	N	M	Mdn	SD	CDC Age Cutoff**	
***CDC-comparable milestones***						
**Began to smile at people**	745	1.77	1.43	2	3	Social
**Started to laugh and/or make squealing sounds**	629	2.3	2.03	2.03	6	Speech
						Social
						Emotional
**Began to bring hands to mouth**	593	1.33	0.73	2.17	4	Fine Motor
						Oral Motor
**Began to babble**	516	2.43	2	2.7	8	Speech
**Began to follow things with eyes**	500	1.57	1.13	3.47	6	Cognitive
**Began to recognize familiar faces**	474	2.17	1.6	2.6	4	Cognitive
						Social
**Shows affection (hugs, cups face, runs to you)**	453	2.6	2	2.73	18	Social
						Emotional
**Began to enjoy looking at himself in a mirror**	436	3.5	3	3.57	6	Social
**Started responding to sounds by making sounds**	423	2.27	1.97	1.93	6	Cognitive
						Speech
						Language Comprehension
**Looking or turning towards sounds**	418	1.57	1.1	3.33	2	Cognitive
						Gross Motor
						Language Comprehension
***Crowd milestones***						
**Newborn reflexive smile**	460	0.87	0.7	0.97		Oral Motor
**Started to roll over from back to tummy** *	352	3.67	3.8	1.63		Gross Motor
**Grasp reflex**	310	0.6	0.13	1.57		Fine Motor
**Started solids**	305	4.6	4.53	1.57		Self Care
						Oral Motor
**Upset when not getting a desired object or activity**	244	5.7	5.5	3.6		Emotional
**Started crawling on belly (commando style)** *	228	5.8	5.8	2.83		Gross Motor
**Started grabbing own legs**	220	4.43	4.13	5.87		Gross Motor
						Fine Motor
**Is now able to grab objects**	184	2.53	2.73	2.1		Fine Motor
**Started clapping hands**	159	6.87	7.1	2.87		Gross Motor
**Started to use a pacifier regularly**	156	1.1	0.53	2.27		Emotional Regulation

Note.

*Milestones with a close CDC version but given their specific endorsement by parents and their developmental difference, were defined as distinct.

**CDC cutoff reflects the last age point by which a milestone should be achieved.

Parents also added to their child’s diary milestone concepts that were not competencies (k = 70; k denotes the number of milestones as opposed to n, which denotes number of children). These milestones had an idiosyncratic nature as in a journal. While they may hold important information for developmental screening, they could not be integrated in the percentile progress reports of the platform. They appeared in a child’s diary but were not suggested to other parents. For research purposes similar non-competence milestones were merged into one concept. These non-competence concepts reflected two categories: (1) Medical (k = 53, 8.22% of database): “First cough” (n = 15), “Has jaundice” (n = 2), “First antibiotics” (n = 2), “First bowel movement” (n = 2). (2) Concerns (k = 17, 2.64% of database): “Has difficulty falling asleep alone” (n = 27), “Refuses to take a bottle” (n = 18), “Trouble burping after feeding” (n = 2), “Doesn’t sit up straight—weak torso” (n = 1). Note that there were another 158 non-competence milestones which were not developmental in nature (e.g., “Brown hair”, “First visit to Church”) and are beyond the scope of this paper (see S1 File for more information).

### Comparison of CDC versus crowd milestone concepts

Fisher’s exact tests were conducted to compare the percentage of crowd versus CDC milestones related to each developmental domain. Results showed a significantly higher rate of CDC versus crowd milestones for the Cognitive (p = .04), Speech (p < .001), and Language Comprehension (p = .04) domains. A significantly larger rate of crowd versus CDC was observed in the Oral Motor (p = .01) and Regulation (p < .001) domains. Fisher’s exact tests showed that there was no significant difference in the rate of a milestone relating to more than one domain in the CDC (44.20%) versus crowd (39.49%) milestones (p = .29).

The most endorsed Regulation milestones authored by the crowd described early sleeping, feeding, and diaper change patterns. These consisted of: “Sleeps for several hours at night (5+)” (Mdn age 3 months), “Shows signs when wants to eat (opens hands, licks hands, looks towards bottle)” (2.6 months), “Rubs eyes when tired” (5.2 months), “Doesn’t cry as much during diaper change” (1.1 months), “More alert during breastfeeding” (1.6 months), “Started asking for a diaper change” (19.9 months), “Started using pacifier when sleeping” (2.3 months), “Started waking up without crying” (3.1 months), “Awake for longer periods during the day” (7.4 months), and “Weaned from nursing” (9.5 months).

The most endorsed Oral Motor crowd milestones were: “Newborn reflexive smile” (Mdn age 0.7 months), “Started feeding from bottle” (0.2 months), “Started to spit up milk” (0.5 months), “Started breastfeeding” (0 months, i.e., birth), “Started to bring pacifier to mouth” (5 months), “Independently eating finger foods (cookie, pear, bread)” (7.2 months), “Started giving kisses” (9.8 months), “Drinks from sippy cup” (7.6 months), “Suck reflex” (0 months, i.e., birth), “Blows raspberries” (4 months).

### Age differences between CDC and crowd milestones

Mann-Whitney U tests indicated that overall, there was a significantly wider age variance reported for CDC comparable milestones (Mdn = 5.2 months) versus crowd milestones (Mdn = 3.73 months) (Z = -4.98, p < .001), with no differences in median age. Next, we examined whether there were age differences within specific developmental domains. Mann-Whitney U tests showed significantly younger median ages of CDC versus crowd milestones in the Speech domain (Z = -2.83, p = .01; Mdn = 13.73, Mdn = 22.52 months, respectively). Significantly higher SD of milestone ages for CDC versus crowd were observed within the Gross Motor (Z = -2.83, p = .005; Mdn = 4.03, Mdn = 3.08 months, respectively), Cognitive (Z = -2.16, p = .03; Mdn = 6.72, Mdn = 4.47 months respectively), and Social (Z = -2.05, p = .04; Mdn = 6.12, Mdn = 4.1 months, respectively) domains.

## Discussion

This study presents the milestones tracked by parents of young children through babyTRACKS, a crowd-based application mediated by experts. Involving parents as active observers of their child’s behavior is valuable for increasing their developmental awareness and partnership with healthcare professionals in early detection of delays, as well as portraying their perceptions of child development. In contrast to a structured checklist, the babyTRACKS platform allows parents to choose when and what to track. Exploring a crowd-driven database of developmental milestones introduces behaviors parents view as important to track and behavioral changes they recognize.

In this open-ended platform, the proportionally higher representation in a diary (above 20%) of major developmental domains (including Gross Motor, Fine Motor, Cognitive and Social domains, which cover core skills) was encouraging in indicating parental awareness of a diverse set of core milestones. Most of the milestones that parents added were comparable to those on existing lists, such as the CDC’s, yet across all 11 developmental domains, they authored “new” milestone concepts that were then adopted by other parents who found them relevant to their own child’s profile. “New” milestones offer an opportunity to update developmental checklists with contemporary milestones—those capturing current societal and cultural contexts. Findings point to differences in domain representations between CDC and crowd milestones, which can guide the refinement of standardized screening procedures to address these gaps and to enhance digital screening efforts to increase parental awareness of milestones they miss.

Previous research indicated that most actions in babyTRACKS were adaptations of existing milestones as opposed to generating semantically new milestone concepts [15], suggesting that authoring “new” milestones does not suit all parents. Authoring milestones requires parents’ active engagement in tracking, which takes more time and effort. A review of idea management in online communities shows that a small proportion of users are active idea generators and even those who are active decline over time [37]. The advantage of obtaining parents’ ideas for milestones is their authentic approach to developmental observations in context. This approach is in line with ecological models of child development [16, 17], which view development as a product of the interaction between a child and the environment. As such, this approach can minimize progress report that is context dependent. By adding milestones that are not contained in traditional screening tools, parents reveal the possible value of detecting new behaviors (if parents notice these behaviors, then they may be important), as well as their reflection of historical changes in the definitions of development. Examples of contemporary milestones added by parents are “started interacting with family dog”, “can use stickers,” and “can operate apps on mom’s phone”. Leading a crowd-based effort based on insights from parents and experts is a promising way to update developmental lists to keep them comprehensive and contemporary.

When parents track behaviors that do not appear in existing tools, such as the CDC list, we learn what is potentially missing from formal screening lists. Among the developmental milestone concepts, only 35% were classified as comparable to those of the CDC. Hence, most of the data introduced new milestone concepts.

Regulation milestones and Oral Motor milestones were mostly introduced by parents as opposed to CDC, and represented very early behaviors. The fact that these domains in babyTRACKS were mainly authored by parents indicates that they are underrepresented in the CDC checklists. Regulation milestones were defined in babyTRACKS as behaviors related to sleep and feeding patterns, diaper change behaviors and self-calming. Oral Motor referred to the use of the mouth in the context of feeding and social interaction. Note that both these domains had an average representation in a dairy below 10%. These findings suggest that parents notice these types of milestones in their daily encounters with their child; however, they are not dominating the dairies. It is also indicative of the parents’ interest in their babies’ feeding, sleeping and contentment.

It is important to detect regulatory problems during infancy early, as they predict later social development [38, 39] and academic achievement [40]. Furthermore, moderate-to-severe regulatory problems are often the only indicators of developmental disorders in infancy, as these disorders become otherwise apparent only later [39]. Nonetheless, Regulation and Social-Emotional milestones are absent from many universal screening tools and developmental checklists for children under the age of 2 years [26, 38]. Regulation is challenging to assess early in life given the rapid changes it undergoes, and its dependence upon environmental factors [40]. There are several baby tracking applications for recording sleep, feeding and diaper change patterns, yet they are not intended to detect development but rather to promote communication between caregivers. Our findings indicate a potential to add another level to such trackers to prompt early detection of atypical regulatory patterns, enabling providers to initiate closer investigation of the sources of dysregulation.

Looking within domains showed that Cognitive, Speech, and Language Comprehension milestones were represented more in CDC than in crowd-authored milestones. Furthermore, in the Speech domain, the CDC milestones had a younger median age (7-month difference) than the crowd milestones did. There was also a narrower age range around crowd milestones. Parents may be less confident in authoring milestones in these domains or are less aware of the nuances of language development (i.e., Speech and Language Comprehension), particularly during the very young ages of pre-verbal communication. For example, capturing the different stages of pre-verbal vocal progress. It is also possible that these domains are sufficiently comprehensive in the CDC checklists, affording less room for new ideas.

Other than speech and language domains, the age cutoff for expected milestones in the CDC checklist are much later than the median ages reported in the babyTRACKS database (see Table 1). This can be attributed to: (1) parents in babyTRACKS reporting the age at which a behavior started as opposed to CDC reporting the latest age at which this point should be achieved, and (2) parents’ interpretation of milestone concepts differed from CDC. However, this age gap is not specific to babyTRACKS, as it was reported in previous research comparing traditional screening tool norms with CDC ages [27].

A very small proportion (if at all) of children’s diaries was devoted to Concerns and Medical information as opposed to reporting developmental progress. The examples of such milestones indicate that parents were not describing severe medical needs and concerns. While not indicating achievements, parental concerns and medical predispositions can have clinical importance for determining a child’s developmental risk [22]. The very low proportion of Concerns and Medical conditions in the database can be associated with the low-risk sample of children and the instructions in the app guiding users to report age-associated progress. The classification of a parent’s text as a concern through the CCI method is not trivial and is reserved for cases in which the parent indicates a concern/delay/difficulty or regression in their child’s behavior. The current design doesn’t enable the integration of this information into a child’s progress report. Integrating features that invite parents to flag their concerns and the crowd of parents and experts to respond to these concerns is a potential path for future design in crowd-based digital screening tools, leveraging the crowd’s wisdom for supporting parents.

Designing a universal, developmental tracking platform attractive to all parents, regardless of referral or the presence of a concern, is challenging. Parents today constantly record and share moments related to their babies’ lives on social media [11, 41, 42]. This is a socially driven phenomenon that is natural to many parents, who report doing so to maintain social relations, obtain social support and feel a sense of a community [42]. Given the importance of involving parents in regular tracking of their child’s development, there are advantages for designing developmental screening platforms that build on natural parenting behaviors. Universal tracking encourages all parents to track milestones and facilitates the collection of rich data on every child, regarding types and timepoints of milestones. A platform that builds on an existing habit of parents is more likely to be integrated into parenting practices and routines.

### Limitations and future research

While crowdsourcing developmental data is advantageous for obtaining both personalized and diverse observations, the data may be biased by not considering the characteristics of the parent or child. For instance, proud parents may report earlier achievements than concerned parents do. The very low rate of concerns in diaries indicate either that the sample was not biased by concerned parents or that concerned parents were reluctant to publicize their child’s delays. In addition, it is important to examine differences app activity and developmental observations of users on parental leave versus full-time working users. Future research is needed to investigate these possibilities.

The internal and external validity checks of these data [13] show promising possibilities for generalization, but further larger-scale studies are needed. Although this study highlights the benefits of engaging parents in all aspects of developmental tracking; expert curation is necessary to ensure non-redundancy and linguistic clarity of concepts. The optimal balance between parent-driven tracking and expert involvement in canonizing parent-authored milestones, as well as limiting the age range and content of the database is yet to be investigated.

The platform was intended for use from birth to age 6 years (see age groups in S1 Table). In practice, half of parents registered when the child was up to 4.2 months old, and the median age of the aggregated milestone concepts was 8.4 months. This may reflect parents noticing the more dramatic developmental changes that occur when a child is closer to one year old, such as moving against gravity (e.g., standing, walking) and being more communicative (e.g., pointing, talking). There is a need to engage parents in facilitating earlier continuous, detailed developmental monitoring which can be critical to obtaining earlier referral, when needed. Collaborating with maternity wards or prenatal care clinics are potential strategies for engaging parents in tracking from birth. Implementing personalized notifications to facilitate milestone recordings of areas in which parents are less observant, can assist in this process.

Although the sample of diary entries was large, very few parents made sequential entries (Mdn of 5 milestones in diary). Continuous use of digital screening platforms has been reported in studies sending a few milestones periodically to prompt low-income mothers to report their children’s development together with providers’ close monitoring of needs [34]. A crowd-based tool builds on parents’ motivation and capacity to track development independently. However, additional mechanisms to engage parents in continuous tracking (e.g., partnering with baby wellness clinics) to obtain richer data and database scale, need to be designed.

Future research should characterize factors that motivate parents to generate new milestones and the impact of authoring milestones upon parenting self-efficacy. Finally, building a platform that enables experts and parents to add new milestones would be an important step toward a true collaborative process of updating checklists while building in voting mechanisms to filter incoming ideas.

## Conclusions

Early childhood development is traditionally assessed by experts relying upon predefined lists of milestones expected for a particular age group. Parents’ increased use of online searches and social media for verifying and sharing information regarding their children offers an opportunity for early digital screening that builds on this habit.

babyTRACKS introduces novel methodologies, as it is an open-ended parent-driven platform that parents embrace. The embedded Crowd-Curated Intelligence (CCI) method adds an important filter for balancing personal and universally-relevant observations to ensure the quality of incoming ideas prior to presenting them to other users. This crowd-based database offers a way to keep developmental lists updated with pertinent, parent-friendly milestones that reflect skills from multiple domains.

Findings showed how parents generated milestones across developmental domains and particularly added more in the regulation and oral motor domains versus their rate in CDC-comparable milestones. Parents should be prompted to track expressive and receptive language milestones, as they are naturally less aware of nuances in their progress, particularly in the younger ages. This study calls for healthcare providers to continue to elicit and listen to parents’ natural observations of their children’s progress.

## Supporting information

S1 Table Examples from babyTRACKS database milestones by age group. (DOCX)

S1 File Other types of milestones. (DOCX)
