# Supplementary material for: The feasibility of a crowd-based early developmental milestone tracking application
Source: PLoS One. 2022 May 26;17(5):e0268548. doi: 10.1371/journal.pone.0268548 (PMC9135273; doi:10.1371/journal.pone.0268548)
Supplement: S1 Table — (DOCX) [file pone.0268548.s001.docx]

# **S1 Table.** **Examples from babyTRACKS database milestones by age group.**

|  | **Mdn age (months)** | **N** | **CDC-comparable milestones** | **Developmental Domain/s** |
| --- | --- | --- | --- | --- |
| **Birth- 2 months** | | | | |
| **Started to use a pacifier regularly** | 0.5 | 156 |  | Emotional  Regulation |
| **Grasp reflex** | 0.1 | 310 |  | Fine motor |
| **Began enjoying taking turns making sounds with parent** | 2 | 342 | X | Speech  Social |
| **Newborn reflexive smile** | 0.7 | 460 |  | Oral motor |
| **Began to follow things with eyes** | 1.1 | 500 | X | Cognitive |
| **2 months- 4 months** | | | | |
| **Is now able to grab objects** | 2.7 | 184 |  | Fine motor |
| **Tries to get things that are out of reach** | 3.2 | 341 | X | Cognitive  Fine motor |
| **Started to roll over from back to** | 3.8 | 352 |  | Gross motor |
| **Started to bring things to mouth** | 2.1 | 397 | X | Fine motor  Oral motor |
| **Started to laugh and/or make squealing sounds** | 2 | 629 | X | Speech  Social  Emotional |
| **4 months- 6 months** | | | | |
| **Started crawling on belly (commando style)** | 5.8 | 228 |  | Gross motor |
| **Upset when not getting a desired object or activity** | 5.5 | 244 |  | Emotional |
| **Started to like playing with others, especially parents** | 4.1 | 248 | X | Social |
| **Plays with objects by shaking, banging, throwing** | 5.3 | 262 | X | Cognitive  Fine motor |
| **Started solids** | 4.5 | 305 |  | Self-care  Oral motor |
| **6 months- 8 months** | | | | |
| **Began to understand "no"** | 7.5 | 142 | X | Cognitive  Language comprehension |
| **Started clapping hands** | 7.1 | 159 |  | Fine motor |
| **Began to play games such as "peek-a-boo" and "pat-a-cake"** | 6.1 | 182 | X | Cognitive  Speech  Social |
| **Started crawling** | 7 | 384 | X | Gross motor |
| **8 months- 10 months** | | | | |
| **Started standing without support** | 9.3 | 181 | X | Gross motor |
| **Started to enjoy playing with other children** | 8.9 | 150 | X | Social |
| **Learned to say several single words** | 10 | 202 | X | Speech |
| **Started using simple gestures, like shaking head for "no" or waving "bye-bye"** | 8.6 | 204 | X | Non-verbal communication  Social |
| **10 months- 12 months** | | | | |
| **Started showing defiant behavior (doing what was told not to)** | 11.2 | 124 | X | Emotional |
| **Started to follow simple, one-step instructions** | 10.4 | 127 | X | Cognitive  Language comprehension |
| **Eating independently with a spoon** | 11.5 | 134 | X | Self-care  Fine motor  Oral motor |
| **Learned to climb up and down from furniture without help** | 11.8 | 145 | X | Gross motor |
| **1 year- 1.5 years** | | | | |
| **Learned how to play simple make-believe games** | 16.2 | 52 | x | Cognitive  Social  Emotional |
| **Names familiar people** | 13.2 | 100 | X | Cognitive  Speech |
| **Began to help undress himself** | 15.8 | 113 | X | Self-care  Gross motor  Fine motor |
| **Started walking independently** | 12.5 | 334 | X | Gross motor |
| **1.5 years- 2 years** | | | | |
| **Climbs up a playground ladder independently** | 20.5 | 32 |  | Gross motor |
| **Started assembling small lego pieces** | 20.8 | 35 |  | Fine motor |
| **Tells us about daily experiences** | 24.3 | 63 | X | Cognitive  Speech  Social |
| **Has complex make-believe play (puts on a show, acts out a story)** | 20.5 | 81 | X | Cognitive  Emotional |
| **2 years- 3 years** | | | | |
| **Asks to go play at a friend's house** | 29.5 | 27 |  | Social |
| **Washes hands alone** | 25.5 | 27 |  | Self-care  Gross motor  Fine motor |
| **Can count higher than 10** | 32 | 32 |  | Cognitive  Speech |
| **Chooses which clothes to wear** | 26 | 49 |  | Emotional  Self-care |
| **3 years- 4 years** | | | | |
| **Can hold breath under water in a pool.** | 37 | 10 |  | Regulation  Oral motor |
| **Began buckling seat belt independently** | 45 | 12 |  | Fine motor |
| **Began to recognize letters** | 47 | 13 |  | Cognitive  Language comprehension |
| **Began drawing people with 6+ body parts** | 37 | 17 | X | Cognitive  Fine motor |
| **4 years- 5 years** | | | | |
| **Learned to play "guess who"** | 53 | 1 |  | Cognitive  Social |
| **Noticed strong heart beats after running** | 51.5 | 3 |  | Cognitive  Regulation |
| **Started riding a two-wheel bicycle independently** | 58 | 4 |  | Gross motor |
| **Started putting on boots with a zipper independently** | 50.6 | 3 |  | Self-care  Fine motor |
| **5 years- 6 years** | | | | |
| **Started to practice tying shoes** | 86 | 1 |  | Fine motor |
| **Read book independently for the first time** | 75 | 1 |  | Cognitive  Language comprehension |
| **Asks others to tell jokes** | 61 | 2 |  | Speech  Social |
| **Started solving math problems** | 62.5 | 3 |  | Cognitive |
