# Supplementary material for: The feasibility of a crowd-based early developmental milestone tracking application
Source: PLoS One. 2022 May 26;17(5):e0268548. doi: 10.1371/journal.pone.0268548 (PMC9135273; doi:10.1371/journal.pone.0268548)
Supplement: S1 File — (DOCX) [file pone.0268548.s002.docx]

# **S2. Other types of milestones**

In addition to the 575 developmental milestones, and the 70 Medical and Concern milestones, there were 158 “Other” milestones recorded. Most of these milestones were recorded within the first six months of life.

The distribution of these “Other” categories with representative examples is as follows:

1. Events (k = 88, 38.60% of non-developmental): “Started tummy time” (n = 86), “Started walking with shoes” (n = 68), “Started having teeth brushed by an adult” (n = 19), “First bath” (n = 16)
2. Physical Features (k = 19, 8.33% of non-developmental): “First tooth” (n = 52), “Weight” (n = 28), “Lost first tooth” (n = 3), “Has a birthmark” (n = 1)
3. Attributes (k = 38, 16.67% of non-developmental): “Often seems happy” (n = 316), “Loves to be in the water” (n = 14), “Very persuasive” (n = 3), “Loves music” (n = 3)
4. Stories (k = 13, 5.70% of non-developmental): “First day at home after being in the hospital, crying and drinking milk” (n = 1), “Met dad for the first time” (n = 1), “Uncle came to visit” (n = 1), “Selfie with the family” (n = 1).

These milestones are idiosyncratic in nature and were added to only 17.77% of diaries. Note that these types of milestones appeared mostly in one diary (67.98%) and some appeared in two diaries (14.47%). Half of the “Other” concepts were recorded under the age of 3.5 months. Users who added “Other” milestones to a child’s diary added significantly more developmental milestones to their child’s diary than those who did not (M = 20.72, SD = 27.73, M = 6.41, SD = 7.70 respectively; t = -24.87, p < .001).

The need of parents to record and share is so strong that this occurred even in a platform such as babyTRACKS that does not invite this behavior (i.e., the platform that does not suggest “Other” milestones in the lists presented to parents), nor does it reward this behavior (i.e., no feedback is provided for these milestones). Parents who add such milestones appear to be more proactive in their digital behavior overall. This notion is supported by our findings that parents who added the “Other” types of milestones tended to add more developmental milestones to their child’s diary. This agrees with previous findings [14] indicating that users who were proactive in solving technological problems and used technology frequently, had a higher positive experience using babyTRACKS. Designing continuous developmental tracking can harness parents’ intensive engagement in documenting every event in their child’s life and sharing it on social media.
